# Dihydroartemisinin remodels macrophage into an M1 phenotype *via* ferroptosis-mediated DNA damage

**DOI:** 10.3389/fphar.2022.949835

**Published:** 2022-08-11

**Authors:** Liu-Gen Li, Xing-Chun Peng, Ting-Ting Yu, Hua-Zhen Xu, Ning Han, Xiao-Xin Yang, Qi-Rui Li, Jun Hu, Bin Liu, Zi-Yi Yang, Xiang Xu, Xiao Chen, Mei-Fang Wang, Tong-Fei Li

**Affiliations:** ^1^ Department of Respiratory, Taihe Hospital of Shiyan, Hubei University of Medicine, Shiyan, China; ^2^ School of Basic Medical Sciences, Hubei University of Medicine, Shiyan, China; ^3^ Hubei Key Laboratory of Embryonic Stem Cell Research, Hubei University of Medicine, Shiyan, China; ^4^ Department of Pathology, Sinopharm DongFeng General Hospital, Hubei University of Medicine, Shiyan, China; ^5^ Department of Pharmacology, School of Basic Medical Sciences, Wuhan University, Wuhan, China; ^6^ School Institute of Chemical Biology and Nanomedicine, State Key Laboratory of Chemo/Biosensing and Chemometrics, College of Chemistry and Chemical Engineering, Hunan University, Changsha, China

**Keywords:** tumor-associated macrophages (TAMs), dihydroartemisinin (DHA), ferroptosis, DNA damage, NF-κB

## Abstract

Lung cancer recruits tumor-associated macrophages (TAMs) massively, whose predominantly pro-tumor M2 phenotype leads to immunosuppression. Dihydroartemisinin (DHA) has been proven to remodel TAM into an anti-tumor M1 phenotype at certain concentrations in the present study, which was hypothesized to facilitate anti-lung cancer immunotherapy. However, how DHA remodels the TAM phenotype has not yet been uncovered. Our previous work revealed that DHA could trigger ferroptosis in lung cancer cells, which may also be observed in TAM thereupon. Sequentially, in the current study, DHA was found to remodel TAM into the M1 phenotype *in vitro* and *in vivo*. Simultaneously, DHA was observed to trigger ferroptosis in TAM and cause the DNA damage response and NF-κB activation. Conversely, the DHA-induced DNA damage response and NF-κB activation in TAM were attenuated after the inhibition of ferroptosis in TAM using an inhibitor of ferroptosis. Importantly, a ferroptosis inhibitor could also abolish the DHA-induced phenotypic remodeling of TAM toward the M1 phenotype. In a nutshell, this work demonstrates that DHA-triggered ferroptosis of TAM results in DNA damage, which could activate downstream NF-κB to remodel TAM into an M1 phenotype, providing a novel strategy for anti-lung cancer immunotherapy. This study offers a novel strategy and theoretical basis for the use of traditional Chinese medicine monomers to regulate the anti-tumor immune response, as well as a new therapeutic target for TAM phenotype remodeling.

## Graphical Abstract

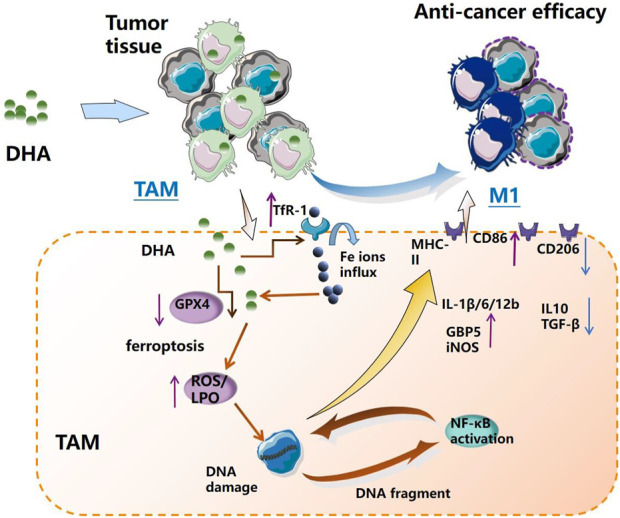

## Introduction

Lung cancer, especially non-small-cell lung cancer (NSCLC), occupies the top three positions in the incidence of malignancies and is the leading cause of mortality in urban cases of cancer (Duma et al., 2019; Yang et al., 2020; Siegel et al., 2022). NSCLC’s microenvironment is characterized by the presence of a large number of tumor-associated macrophages (TAMs), which display an immunosuppressive M2 phenotype (Li et al., 2018; Hwang et al., 2020). TAM interacts with lung cancer cells and various types of immunocytes (Singhal et al., 2019; Wei et al., 2019), promoting the proliferation of malignant cells and repressing the anti-tumor immune response (Lavin et al., 2017). This has resulted in lung cancer being difficult to be completely destroyed and highly susceptible to recurrence and metastasis. However, the phenotype of TAM can be reprogrammed to an immune-excitable, anti-tumor M1 phenotype under certain conditions (Li et al., 2017; Parayath et al., 2018; Osipov et al., 2019; Lu et al., 2020; Sarode et al., 2020; Xu et al., 2021; Yu et al., 2021). Therefore, remodeling the phenotype of TAM has become a crucial strategy of great interest in immunotherapy of lung cancer.

Dihydroartemisinin (DHA) is a multipotent chemotherapeutic agent (Keating, 2012; Tu, 2016; Dai et al., 2021). In addition to its ability to destroy plasmodium, DHA has been investigated for its cytotoxic effects on malignant cells of various solid tumors, including esophageal cancer, breast cancer, and glioma (Kumar et al., 2019; Liu et al., 2019; Wan et al., 2019). However, few studies are concerned with the effect of DHA on immunocytes. It is reported that DHA can induce ferroptosis by down-regulating glutathione (GSH) and glutathione peroxidase 4 (GPX4), and promoting the intracellular accumulation of reactive oxygen species (ROS) and lipid peroxide (LPO) (Chen et al., 2020; Yi et al., 2020). Our previous work also revealed that DHA uptake by lung cancer cells effectively induced apoptosis. More importantly, the transferrin-1 (TfR1) and cyclooxygenase-2 (COX-2) expression were up-regulated along with an intracellular accumulation of LPO, but the GPX4 expression was decreased (Han et al., 2022). Based on the aforementioned, DHA is likewise able to trigger the occurrence of ferroptosis in TAM. DHA generates certain concentrations of ROS and LPO in TAM by triggering ferroptosis, which results in oxidative damage to the DNA of TAM. Supposedly, interferon-stimulating molecules (STING) in the cytoplasm have been identified to be activated by fragments of damaged DNA, which in turn revitalizes its downstream NF-κB signaling pathway (Hou et al., 2018; Yum et al., 2021). This inflammatory signaling, on the other hand, can polarize macrophages into a pro-inflammatory, anti-tumor M1 phenotype (Lin et al., 2020; Yu et al., 2022).

DHA showed a significant remodeling effect on mouse primary macrophages toward the M1 phenotype. Similarly, macrophage infiltration and phenotypic remodeling were enhanced in the tumor tissues of DHA-treated lung cancer cell-bearing mice. Further studies indicated that DHA treatment also initiated ferroptosis, DNA damage, and NF-κB activation in TAM. But the inhibition of ferroptosis in TAM significantly attenuated the DHA-induced DNA damage and NF-κB activation. Synchronously, the inhibition of ferroptosis also weakened the phenotypic remodeling of TAM by DHA. The current study for the first time uncovered the mechanism by which DHA reprograms macrophages through ferroptosis, providing a novel approach for the intervention in TAM phenotypic remodeling.

## Material and methods

### Cell models and DHA treatment

According to a previous study (Xu et al., 2021), mouse bone marrow-derived cells were extracted from C57/BL mice at 4–5 weeks of age (Laboratory Animal Center at the Hubei University of Medicine, Hubei, China). Macrophage colony-stimulating factor (M-CSF) was applied to induce mouse bone marrow-derived cells into mouse bone marrow-derived macrophages (mBMDMs). Upon this foundation, IL-4 and IL-13 were added to the mBMDM for 2 days to promote its polarization to M2, yielding the mouse TAM (Supplementary Figure S1). Alternatively, Lewis cells were utilized as a model for mouse lung cancer cells. Lewis cells were purchased from the Cell Bank of Shanghai Institutes for Biological Sciences (Shanghai, China). All cells were cultured in DMEM (Sigma-Aldrich, St Louis, United States) supplemented with 10% fetal bovine serum (QmSuero/Tsingmu Biotechnology, Wuhan) in an incubator with humidification (5% CO_2_/95% air ambience, 37°C). DHA (77,939-50-9, MERYER, Shanghai, China, 98% purity) was applied to treat TAM for about 18 h. The concentration of DHA used in this work ranged from 5 μg/ml to 60 μg/ml.

### TAM viability detection

In order to evaluate the efficacy, TAMs were treated with DHA for 18 h. TAMs were plated in 96-well plates with a density of 6 × 10^3^ per well treated with DHA (different concentrations) for 18 h. The cell viability was detected using a CCK-8 Kit (HY-K0301, MCE, NJ, United States).

### Phenotype assay of macrophages

DHA-treated TAMs were harvested, and then marked with fluorescein-linked CD206 and CD86. The surface expression of CD206 and CD86 was detected by flow cytometry. In addition, MHC-II expression was assayed through immunofluorescence staining and observed with microscopy. Alternatively, proteins of TAM were harvested. The expression of iNOS and GBP5 was assayed by Western blotting (WB). Furthermore, phagocytosis function was analyzed by the incubation of latex beats. The activated latex beats were incubated with DHA-treated TAMs for 2 h. Cells were washed with PBS three times and fixed using 4% paraformaldehyde, and then labeled with Hoechst 33,342. The latex beats in mBMDM were detected using laser scanning confocal microscopy (LSCM). Moreover, IL-6 and IL-1β expression of mRNA were detected by a real-time PCR (RT-PCR). Finally, the release of IL-6 and IL-1β was measured with Elisa kits (CME0006/CME0015, purchased from 4A Biotech Co., Ltd.).

### ROS detection

TAMs were seeded in 24-well plates with a density of 3×10^5^ cells per well, and then incubated with DHA for 18 h. The cells were then washed with PBS three times and incubated with 10 μM of 2,7-dichlorodi-hydrofluorescein diacetate (DCFH-DA, S0033, Beyotime, Shanghai, China) at 37°C for 40 min before being harvested and assayed by flow cytometry.

### Observation of ferroptosis

For the assay of TAM’s ferroptosis, first intracellular Fe ions were investigated. In brief, the PGSK probe was applied to label TAM for 15 min. After that, the fluorescent dye was removed. The cells were washed with PBS three times. The FITC fluorescence of PGSK-labelled TAM was analyzed through flow cytometry. Since the PGSK probe can be quenched by iron ions, lower intracellular fluorescence indicates a higher iron ion concentration. Moreover, malondialdehyde (MDA) production, which is an indicator of ferroptosis, in TAM was detected using an MDA Assay Kit (E-BC-K025-S, Elabscience).

### DAN damage detection

DNA double-strand breaks (DDSBs) were detected by the comet assay (Olive and Banáth, 2006). In brief, TAMs were seeded in 24-well plates and treated with DHA. Cells in PBS were prepared and mixed with a low melting point agarose. The mixture was then dripped onto a glass slide pre-coated with agarose gel and pressed, followed by electrophoresis at 20 V, 250 mA for 20 min. The mixture was then neutralized using tris-Hcl (PH = 6.0) for 30 min. Finally, Hoechst 33,342 was used to stain the nuclei. The cells were photoed using fluorescence microscope. For DNA damage response analysis, TAM in the 6-well plates were treated with DHA. Then total proteins were extracted for WB assay of γ-H2A.X and p53 expression.

### Assay of activation of NF-κB

TAMs were seeded in 6-well plates or special dishes, and treated as mentioned previously. First, the expression of NF-κB, p-NF-κB was detected by WB. Alternatively, the nuclear translocation of NF-κB was analyzed through immunofluorescent staining of NF-κB and LSCM.

### Inhibition of ferroptosis

For the inhibition of TAM’s ferroptosis, ferrostatin-1 (Fer-1, 2 μM) was applied to treat TAM along with DHA for 16–20 h. The ferroptosis of TAM was evaluated following the blockage to confirm the successful blockage.

### Measurement of anti-tumor efficacy

To examine the anti-tumor efficacy of DHA-treated TAM, Lewis lung cancer cells (LLC) were co-cultured with TAM with or without DHA treatment for another 24 h. The LLC could be harvested based on the different attachment capacities. The obtained cells were stained with Annexin-V/PI (purchased from CHAMOT BIOTECHNOLOGY CO., LTD.), and then analyzed by flow cytometry. Alternatively, the cell viability of LLC was detected using the CCK-8 Kit as described previously. At the same time, proteins of LLC were extracted to analyze the expression of apoptosis-associated molecules.

### Immunofluorescent staining of CD86, CD206, NF-κB, and MHC-II

For the detection of surface expression of MHC-II or the location of NF-κB, cells were incubated with primary antibodies of CD86 (212927, Elabscience, China), CD206 (B354282, Biolegend, United States), NF-κB (10745-1-AP, Proteintech, Wuhan, China), and MHC-II (sc-66205, SANTA CRUZ BIOTECHNOLOGY, Santa Cruz, America) overnight at 4 C, and then incubated with goat anti-rat IgG/Alexa fluor 488 secondary antibodies (bs-0293G-AF488, Bioss, Beijing, China) for another 120 min. After incubating with fluorescent secondary antibodies, the cells were washed three times before flow cytometry or LSCM.

### Flow cytometry detection

Fluorescence of FITC-Annexin-V, DCFH-DA, C11-BODIPY-FITC, and Alexa fluor 488 was acquired in the FITC channel. PI and C11-BODIPY-PE fluorescence was acquired in the PE channel. The excitation wavelength and emission wavelength were 488 nm and 525 nm in the FITC channel, 561 nm and 585 nm in the PE channel, respectively. After cells were processed and collected as described previously, they were filtered into special tubes for flow cytometry. Each channel was adjusted to the appropriate voltage before collecting cells. At least 1 × 10^4^ cells per sample were acquired for every collection. Geometric means (GM) were used to quantify the mean fluorescent intensity (MFI).

### Western blotting

TAM treated by DHA or other controls were washed three times with PBS and lysed in a RIPA buffer with 1% protease inhibitor for 40 min. Cell lysates were centrifuged and protein concentration was measured using a BCA Assay Kit. Equal proteins (10–30 μg) were fractionated by SDS-PAGE and transferred to a PVDF membrane. The membranes were blocked with 3–5% bovine serum albumin in TBST and incubated with primary antibodies of iNOS (ab15323, Abcam, Cambridge, United Kingdom), GBP5 (13220-1-AP, Proteintech, Wuhan, China), p53 (bs-2090R, Bioss, Beijing, China), γ-H2A.X (bs-3185R, Bioss, Beijing, China), NF-κB (10745-1-AP, Proteintech, Wuhan, China), p-NF-κB (bs-0982R, Bioss, Beijing, China), and GAPDH (PMK053C, BioPM, Wuhan, China) overnight at 4°C. Then the membranes were incubated with horseradish peroxidase-conjugated secondary antibodies. Protein bands were reacted using an ECL kit (PMK003, BioPM, Wuhan, China), and the films were exposed using a Bio-Imaging system (170-8265, Bio-Rad).

### RT-PCR assay

mRNA of TAM was extracted and reverse-transcribed to cDNA, which was amplified using an SYBR Green qPCR Master Mix kit (PC3301, Beijing, Aidlab). RT-PCR was performed using a Bio-RAD CFX Connect Optics Module and data were analyzed using Bio-RAD CFX Manager. The specific primer sequences are as follows:

Mouse interleukin-1β (IL-1β) forward: AGC​TCC​CTT​TTC​GTG​AAT​GAG​CAG​A
Mouse IL-1β reverse: ATG​GTT​TCT​TGT​GAC​CCT​GAG​CGA​C
Mouse IL-12b forward: ATG​AAG​GAG​ACA​GAG​GAG​GGG​TGT​A
Mouse IL-12b reverse: TGC​TGC​ATG​AGG​AAT​TGT​AAT​AGC​GA
Mouse cyclooxygenase-2 (COX-2) forward: TGA​GTA​CCG​CAA​ACG​CTT​CT
Mouse COX-2 reverse: CTC​CCC​AAA​GAT​AGC​ATC​TGG
Mouse IL-6 forward: CGG​AGA​GGA​GAC​TTC​ACA​GAG.
Mouse IL-6 reverse: ATT​TCC​ACG​ATT​TCC​CAG​AG.
Mouse glutathione peroxidase 4 (GPX4) forward: TTC​AGC​TCA​GGG​ATG​ACC​TT
Mouse GPX4 reverse: CCT​CCA​TGG​GAC​CAT​AGC​GCT​TC
Mouse transferrin-1 (TfR1) forward: CTG​GCT​CTC​ACA​CTC​TCT​CAG​CTT​T
Mouse TfR1 reverse: GCA​TTT​GCG​ACT​CCC​TGA​ATA​GTC​C
Mouse IL-10 forward: AGC​TCC​AAG​ACC​AAG​GTG​TCT​ACA​AG
Mouse IL-10 reverse: AGT​CCA​GCA​GAC​TCA​ATA​CAC​ACT​G
Mouse transforming growth factor-β (TGF-β) forward: CCT​GCC​CCT​ATA​TTT​GGA​GCC​TGG​A
Mouse TGF-β reverse: GTA​GTA​GAC​GAT​GGG​CAG​TGG​CTC​C
Mouse GAPDH forward: AGG​TCG​GTG​TGA​ACG​GAT​TTG
Mouse GAPDH reverse: TGT​AGA​CCA​TGT​AGT​TGA​GGT​CA

### Immunohistochemistry assay in lung cancer cell-bearing mice

Female C57BL/6 mice at 5–6 weeks of age (18∼20 g) were housed in the Animal Center at the Hubei University of Medicine (Hubei, China) with a temperature-controlled environment, fresh water, and rodent diet. Animal handling and experimental procedures were in line with protocols approved by the Animal Care Committee at the Hubei University of Medicine. All inoculations and treatments were carried out under Nembutal anesthesia. To investigate the effects of DHA on the ferroptosis, DNA damage, NF-κB activation, and phenotype of TAM, LLC-bearing mice received DHA treatment. For about 24 h after treatment, the mice were sacrificed. The tumor grafts were harvested. Fresh tissues of neoplasm were crushed and assayed for their internal iron ions. Paraffin sections of neoplastic tissue were dewaxed, rehydrated, and antigen-repaired with sodium citrate for 15 min. Then the paraffin sections were subjected to incubation in 3% hydrogen peroxide at room temperature for 15 min. Paraffin sections were blocked with 5% BSA for 60 min, dyed with primary antibodies overnight at 4°C, and then stained with secondary antibody (PV-9000, ZSGB-BIO, Beijing, China) for 1 h at 37°C. Diaminobenzidine (DAB, ZL-9018, ZSGB-BIO, Beijing, China) was used for staining at room temperature for 1–3 min. The nuclei were stained with hematoxylin or DAPI. Primary antibodies included CD11b (66519-1-Ig, Proteintech, Wuhan, China), F4/80 (E00611-1637, eBioscience, United States), iNOS (ab15323, Abcam, Cambridge, United Kingdom), GBP5 (13220-1-AP, Proteintech, Wuhan, China), MHC-II (sc-66205, SANTA CRUZ BIOTECHNOLOGY, Santa Cruz, United States), GPX4 (14432-1-AP, Proteintech, Wuhan, China), COX-2 (A1253, Abclonal, Wuhan, China), γ-H2A.X (bs-3185R. Bioss, Beijing, China), p53 (bs-2090R, Bioss, Beijing, China), and NF-κB (10745-1-AP, Proteintech, Wuhan , China). Last, observation of paraffin sections was performed with an orthogonal Olympus microscope or confocal microscopy.

### Statistical analysis

All statistics were shown using the mean ± standard deviation (SD). Statistical differences between the groups were analyzed by one-way analysis of variance (ANOVA). Statistical differences in Figures 2B,C and Figures 4B,C were analyzed through Student’s t-test. *p* values < 0.05 were considered to be statistically significant.

## Results

### DHA showed outstanding ability for phenotypic remodeling of macrophages toward M1

**FIGURE 1:**
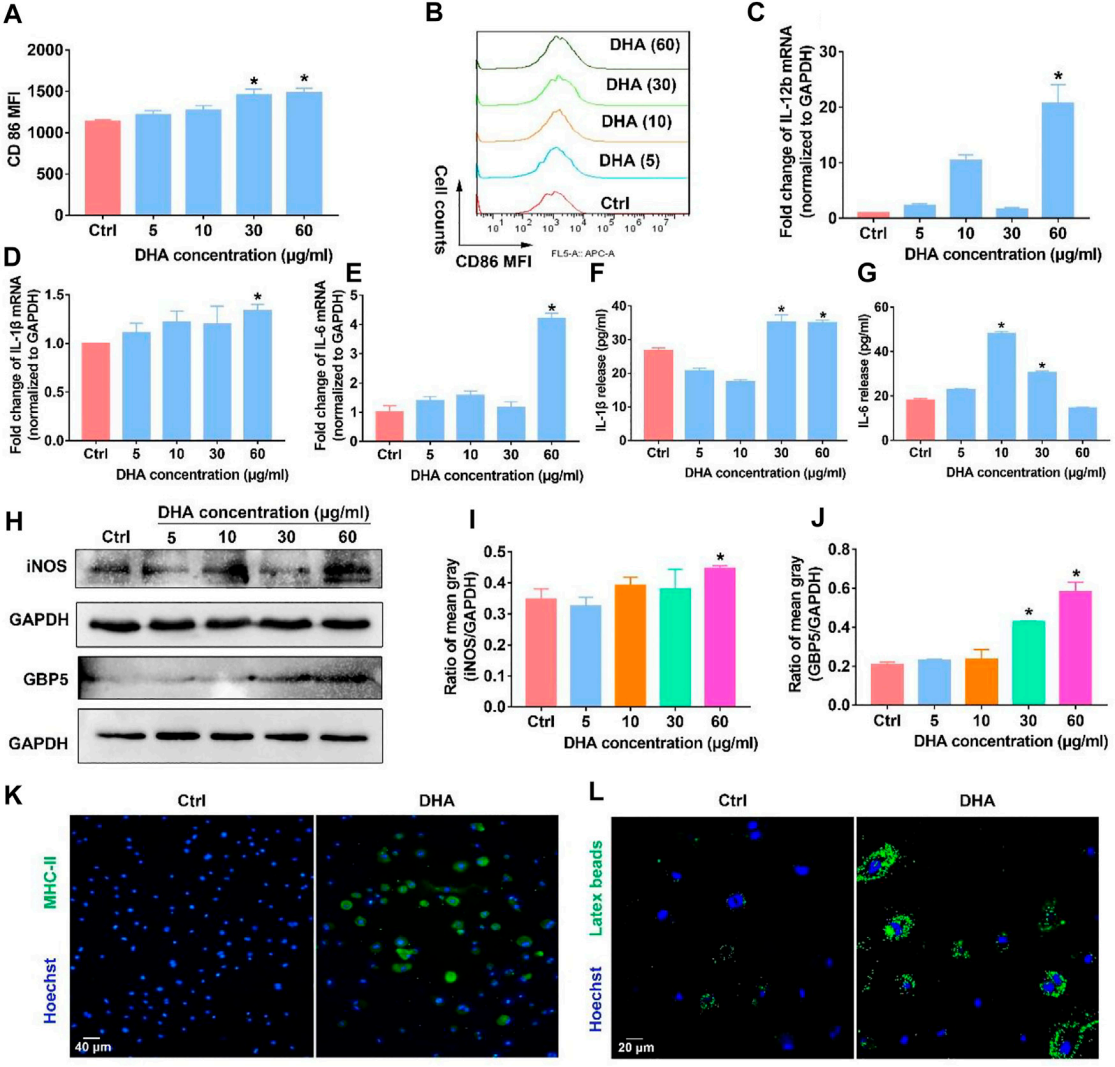
DHA remodeled macrophages into the M1 phenotype *in vitro*. **(A,B)** The membrane expression of CD86 was assayed through flow cytometry. **(C–E)** mRNA expression of IL-1β, IL-6, and IL-12b was investigated using the RT-PCR. **(F,G)** Release of IL-1β and IL-6 was detected using the Elisa Kit. **(H–J)** WB was applied to measure the expression of iNOS and GBP5. The mean gray was quantitatively analyzed. **(K,L)** The functions of antigen presentation and phagocytosis were detected, as evidenced by MHC-II expression and latex beats experiments. Geometric means were used to quantify the MFI. Values were means ± SD (*n* = 3, *p < 0.05 when compared with the control group).

**FIGURE 2:**
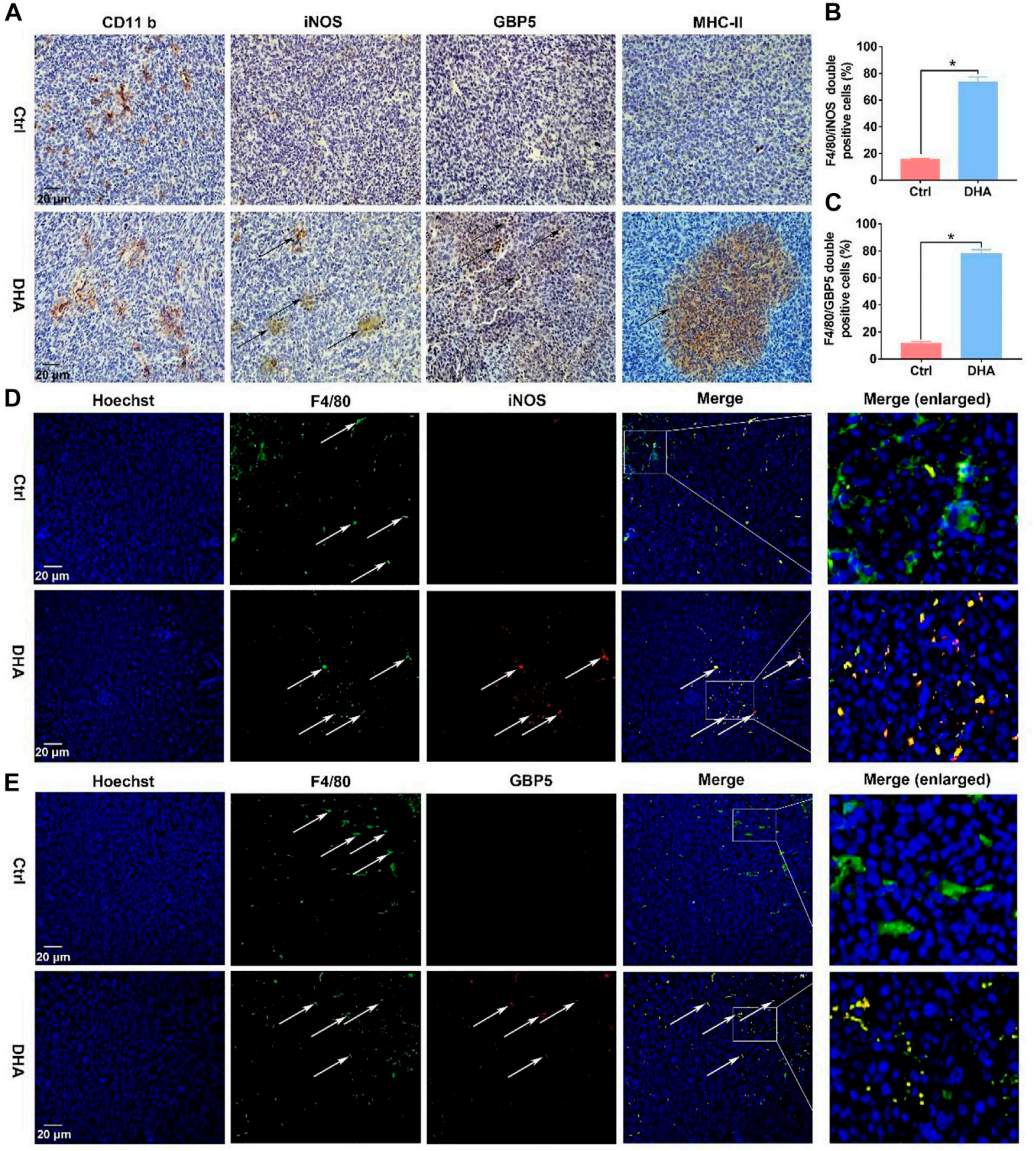
DHA reprogrammed macrophages into the M1 phenotype in tumor tissues. **(A)** The expression of CD11b, which is a biomarker of macrophages, was assayed by IHC. In addition, M1 phenotypic molecules, such as iNOS, MHC-II, and GBP5, were detected through IHC as well. **(B,C)** F4/80/iNOS and F4/80/GBP5 double-positive cells in the tumor grafts were counted. **(D,E)** The infiltration and phenotypic remodeling of macrophages were assayed by multiple immunofluorescent staining of F4/80 and iNOS, GBP5. Green fluorescence came from F4/80. Red fluorescence came from iNOS or GBP5. Yellow fluorescence represented F4/80/iNOS or F4/80/GBP5 double-positive cells (type-I macrophages). Values were means ± SD (*n* = 3, *p < 0.05 when compared with the control group).

Before exploring DHA’s effect on the phenotypic remodeling of TAM, the viability of macrophages was investigated first. The viability of TAM varied little in the presence of DHA as presented in Supplementary Figure S2. Subsequently, as shown in Figure 1, the phenotype of the macrophage was assayed. The biomarkers of the M1 phenotype, surface CD86, were up-regulated synchronously (Figures 1A,B). In addition, the expression of IL-1β, IL-6, and IL-12b, which are crucial pro-inflammatory cytokines, increased in DHA-treated TAM (Figures 1C–E). In agreement with the mRNA expression, the release of IL-1β and IL-6 was also elevated in the presence of DHA (Figures 1F,G). Moreover, the results of iNOS and GBP5 expression showed significant regulation in macrophages treated by DHA (Figures 1H–J). Furthermore, the obvious M1 phenotype transformation of DHA-treated macrophages was indicated by the enhanced functions of antigen presentation and phagocytosis, characterized by the enhanced expression of MHC-II and endocytic latex beats of TAM (Figures 1K,L). Interestingly but not surprisingly, the indicators of M2 phenotype such as CD206, IL-10, and TGF-β were down-regulated in response to DHA, suggesting depolarization of TAM (Supplementary Figure S3). Consistent with the *in vitro* findings, there was obvious macrophage infiltration in the tumor grafts of mice that received DHA treatment, as suggested by the significant CD11b expression. Importantly, the tumor tissue of mice treated with DHA showed enhanced iNOS, GBP5, and MHC-II expression, indicating the M1 reprogramming of TAM inside the tumor tissue (Figure 2A). In addition, more F4/80/iNOS or F4/80/GBP5 double-positive cells could be observed in the tumor grafts of mice treated by DHA (Figures 2B–E). The findings of co-staining of F4/80, GBP5, and iNOS were crucial evidence that DHA remodels TAM into an M1 phenotype in tumor tissues. In a word, TAM treated with DHA exhibited apparent M1 phenotype reprogramming.

### DHA induced ferroptosis of macrophage

**FIGURE 3:**
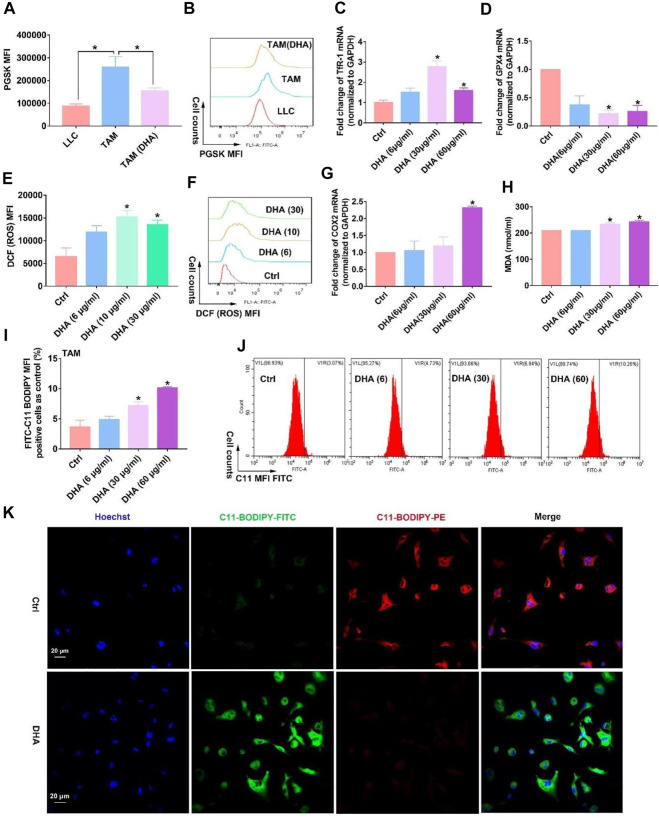
DHA treatment of TAM resulted in the accumulation of iron ions and inhibition of GPX4l, thus generating ROS and LPO and inducing ferroptosis *in vitro*. **(A,B)** Intracellular iron ions were assayed using the PGSK probe and flow cytometry. **(C,D)** TfR-1 and GPX4 mRNA expression was analyzed by the RT-PCR. **(E,F)** ROS generation in TAM was detected by the DCFH-DA probe. **(G)** COX-2 mRNA expression was analyzed by RT-PCR. **(H)** MDA concentration in TAM was measured using an MDA Assay Kit. **(I,J)** C11-BODIPY fluorescent dye was applied to detect LPO production wherein FITC fluorescence-positive cells represented accumulated LPO. **(K)** Cellular LPO generation was observed by confocal microscopy. Geometric means were applied to quantify the MFI. Values were means ± SD (*n* = 3, *p < 0.05 when compared with the control group).

**FIGURE 4:**
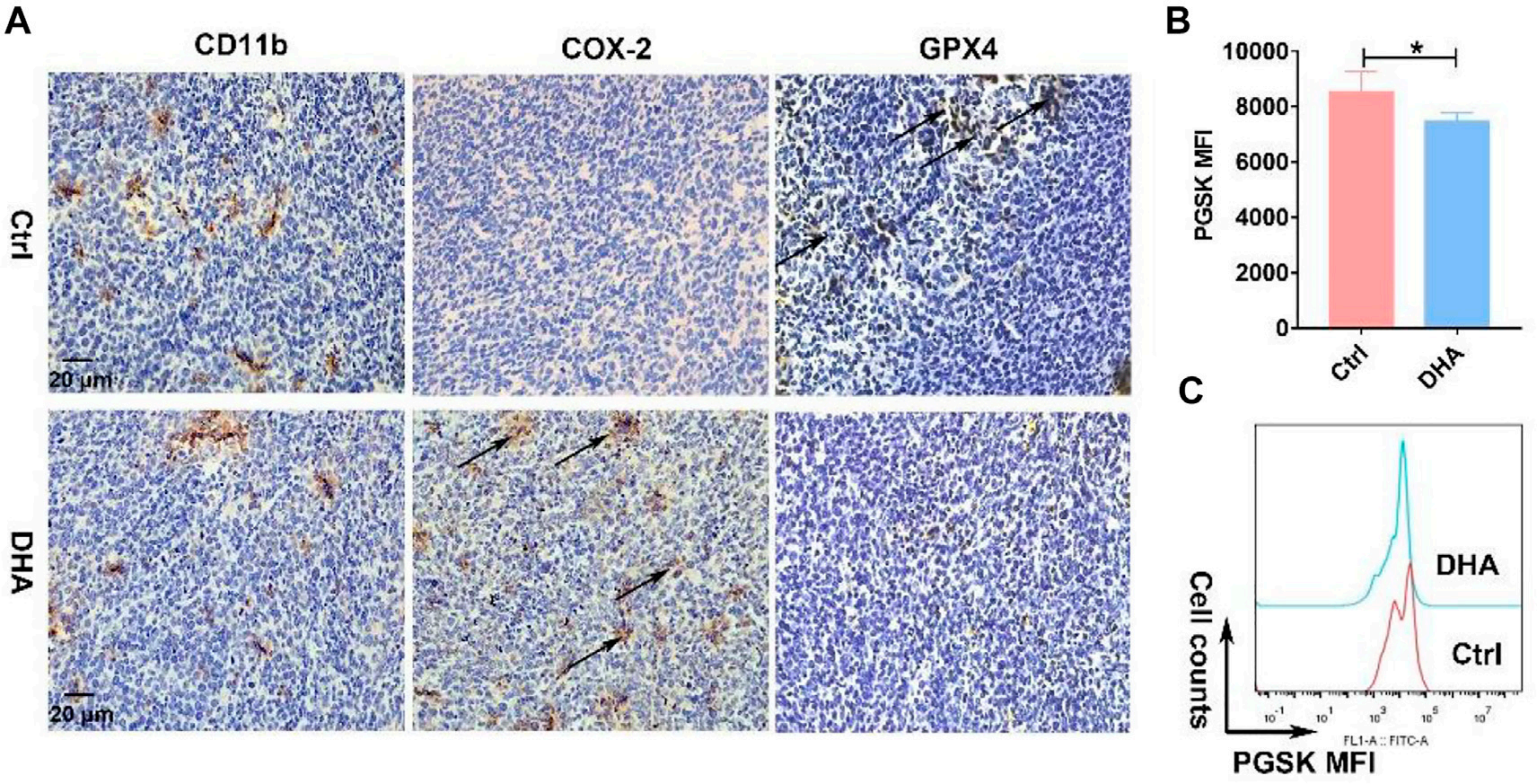
DHA activated ferroptosis *in vivo*. **(A)** Obvious expression of CD11b in the tumor grafts indicated the infiltration of macrophages. Induction of macrophages’ ferroptosis, which was evidenced by prominent COX-2 and reduced GPX4 expression, was analyzed by IHC staining. **(B,C)** Iron ions in the tumor grafts were measured using the PGSK probe. Decreased fluorescence of PGSK indicated elevated iron ions. Geometric means were applied to quantify the MFI. Values were means ± SD (*n* = 3, *p < 0.05).

Elucidating how DHA regulates TAM for the M1 phenotype is of critical interest for understanding the pharmacological effects of DHA. With this concern in mind, we first explored what happened upstream after the action of DHA on TAM. As mentioned previously, given that DHA can induce ferroptosis in lung cancer cells, did the action on TAM trigger the ferroptosis as well? As expected, the iron ions in TAM were significantly lower than those in lung cancer cells. However, DHA treatment promoted the accumulation of iron ions (Figures 3A,B), which may be associated with the up-regulation of TfR1 (Figure 3C). The PGSK probe is a common reagent for the detection of cellular iron ions, whose fluorescence can be quenched by iron ions in living cells. Thus, low fluorescence implied a higher concentration of iron ions in TAM. As reported, TfR1 contributes to the influx of iron ions in most cells. In addition to increased iron ions, the represented molecule of the reducing system GPX4 in TAM was down-regulated in the presence of DHA (Figure 3D). We then investigated the cellular ROS and LPO generation. As shown in Figure 3 e-f and i-k, after incubation with DHA, increased DCFH-DA fluorescence and FITC-C11-BODIPY positive cells in TAM were observed, indicating the accumulation of ROS and LPO. In addition, the expression of COX-2 and MDA accumulation, biomarkers of ferroptosis, up-regulated in response to DHA as well (Figures 3G,H). Notably, the ROS generation and up-regulation of TfR-1 were more significantly at 30 μg/ml than 60 μg/ml, which may be associated with the decreased viability of TAM. The *in vivo* results also displayed stronger ferroptosis in lung cancer cell-bearing mice that received DHA treatment, as indicated by the augmented expression of COX-2 and reduced expression of GPX4 (Figure 4A). Furthermore, in agreement with the *in vitro* data, the iron ions were elevated in mice treated with DHA (Figures 4B,C). These findings strongly suggest that DHA promoted iron ion enrichment and inhibited GPX4, thereby generating ROS and LPO, resulting in the induction of ferroptosis in macrophages.

### DHA-treated TAM exhibited enhanced DNA damage and activation of NF-κB

**FIGURE 5:**
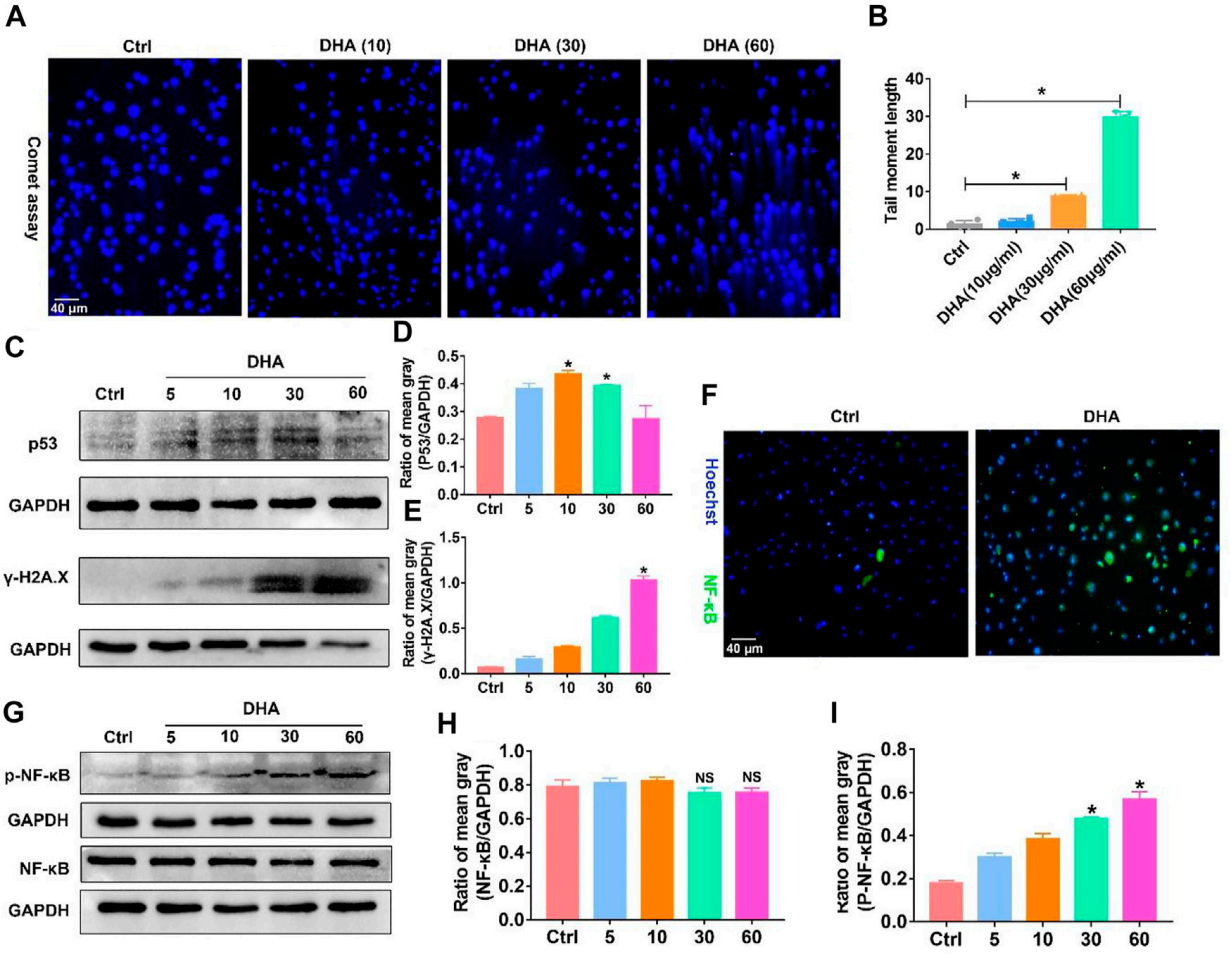
TAM treated with DHA showed prominent DNA damage and activation of NF-κB *in vitro* and *in vivo*. **(A,B)** The DNA double-strand break was detected through the comet assay. The comet tail length was counted. **(C–E)** The expression of p53 and γ-H2A.X, which are biomarkers of the DNA damage response, was investigated using WB. The mean gray was quantitatively assayed. **(F)** The nuclear-translocation of NF-κB was observed by immunofluorescent staining. Green fluorescence came from NF-κB. **(G–I)** WB results revealed the phosphorylation of NF-κB, as indicated by the enhanced expression of p-NF-κB. NF-κB expression was also measured as control. The mean gray was quantitatively assayed. Values were means ± SD (*n* = 3, *p < 0.05 which was compared with the control group).

**FIGURE 6:**
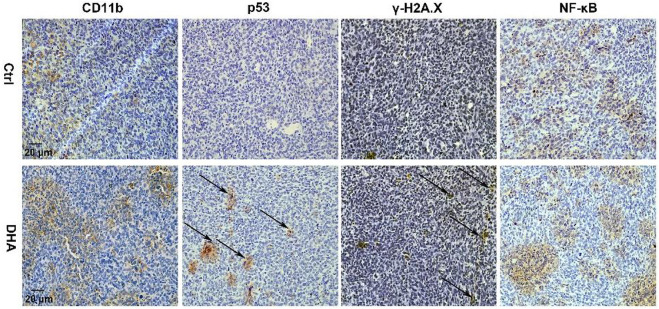
DHA facilitated DNA damage and NF-κB activation in lung cancer cell-bearing mice. IHC staining suggested DHA treatment significantly increased the expression of p53, γ-H2A.X, and NF-κB of TAM in tumor grafts.

The generated ROS and LPO accompanied with the ferroptosis of TAM possess the function of attacking DNA, causing DNA damage such as DNA double-strand break (DDSB). Therefore, the DNA fragments and subsequent DNA damage response (DDR) in TAM treated with DHA were investigated. The visible but not very serious DDSB could be monitored in the DHA treatment group with the comet assay (Figures 5A,B). Along with that, the DDR in TAM treated with DHA was observed, as indicated by the enhanced expression of γ-H2A.X and p53 (Figures 5C–E). Furthermore, DHA-treated TAM showed significant activation of NF-κB, which was characterized by phosphorylation and nuclear-translocation of NF-κB (Figures 5F–I). The *in vivo* results were in agreement with *in vitro* findings. As presented in Figure 6, the DNA damage and activation of NF-κB were demonstrated in the tumor grafts of DHA-treated mice, as evidenced by the enhanced expression of γ-H2A.X, p53, and NF-κB detected by IHC. Since NF-KB is a downstream signal of DNA damage, it could lead to the inflammatory reaction of TAM. There is speculation that this may be related to the M1 phenotypic remodeling of macrophages. Taken together, TAM treated with DHA exhibited prominent DNA damage and NF-κB activation. Based on this proof, we speculated that DHA-mediated ferroptosis in TAM produced adequate ROS and LPO, resulting in DNA damage and subsequent DDR to activate the NF-κB molecule. TAM were polarized into pro-inflammatory phenotype (M1) by the activated NF-κB signaling pathway thereupon.

### Blockage of ferroptosis impaired DHA-mediated DNA damage and reprogramming in macrophages

**FIGURE 7:**
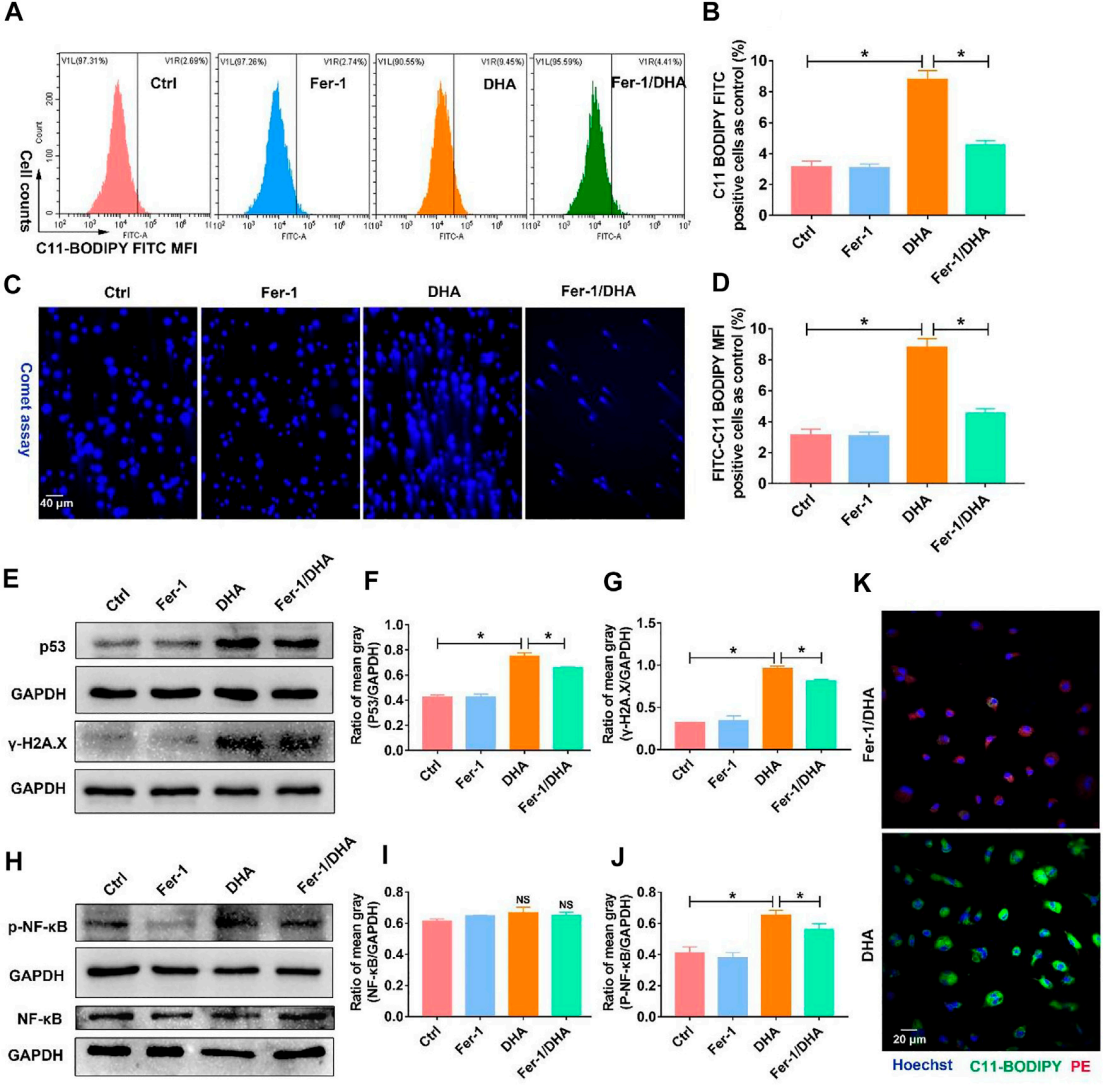
Inhibition of ferroptosis using Fer-1 reduced the DNA damage and activation of NF-κB triggered by DHA. **(A,B)** The LPO generation, which reflected the ferroptosis of TAM, was assayed using the C11-BODIPY probe. The cellular LPO fluorescence (FITC) was measured through flow cytometry. **(C,D)** The DDSB was assayed by comet experiments. The comet tail length was counted. **(E–G)** The expression of p53 and γ-H2A.X, and comet assay were detected to confirm the degree of DNA damage. The results were quantitatively analyzed using mean gray. **(H–J)** Activation of NF-κB was assayed through the expression of p-NF-κB in TAM. The results were quantitatively analyzed using the mean gray. **(K)** The LPO generation, which was assayed using the C11-BODIPY probe, was observed by confocal microscopy. Green fluorescence represented LPO accumulation. Values were means ± SD (*n* = 3, *p < 0.05).

In order to establish the logical link among DHA-induced ferroptosis, DNA damage, and phenotypic remodeling in TAM, ferrostatin-1 (Fer-1), the LPO scavenger, was applied to inhibit the TAM’s ferroptosis. Then the DNA damage mediated by DHA was accessed again. First, the total LPO accumulation in DHA-treated TAM was reduced with the addition of Fer-1 (Figures 7A,B,K). DDSB caused by DHA was also attenuated by the co-addition of Fer-1 (Figures 7C,D). Moreover, as displayed in Figures 7E–G, the expression of γ-H2A.X and p53 was reduced in the presence of Fer-1 and DHA compared with DHA treatment alone, suggesting that the inhibition of ferroptosis could impair the DHA-induced DNA damage in TAM. Meanwhile, DHA-activated NF-κB was abated by co-treatment with Fer-1, as indicated by the decreased expression of phosphorylated NF-κB (Figures 7H–J and Supplementary Figure S4). Based on these results, the inhibition of ferroptosis in TAM was demonstrated to weaken the DHA-induced DNA damage and activation of NF-κB.

**FIGURE 8:**
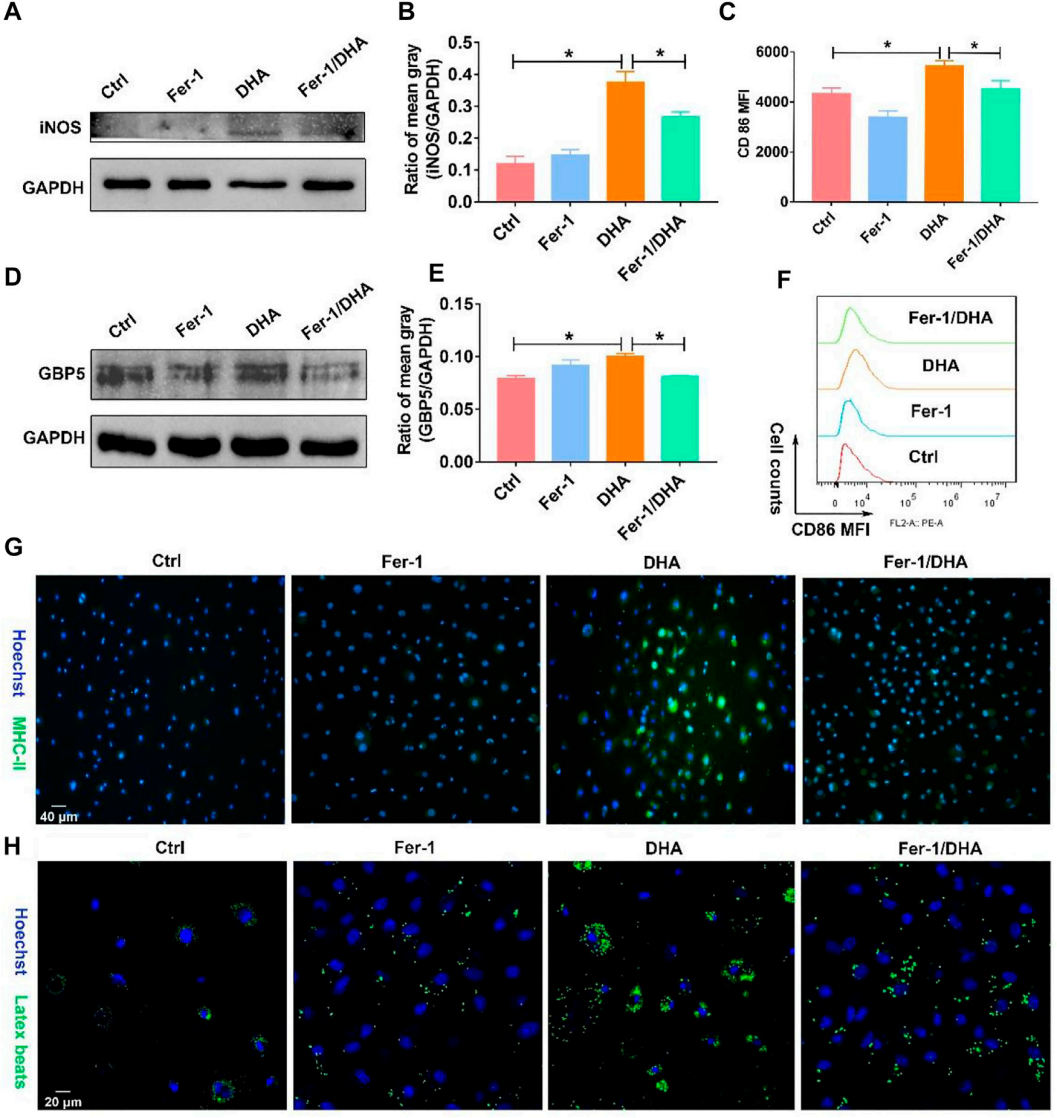
Fer-1 suppressed the remolding effect of TAM toward the M1 phenotype induced by DHA. **(A,B)** iNOS expression in TAM was measured using WB. The mean gray was quantitatively analyzed. **(D,E)** GBP5 expression in TAM was measured using WB. The mean gray was quantitatively analyzed. **(C–F)** The CD86 expression of TAM was analyzed through flow cytometry. **(G,H)** MHC-II immunofluorescent staining and latex beats assay were applied to demonstrate the antigen presentation ability and phagocytosis function. Geometric means were used to quantify the MFI. Values were means ± SD (*n* = 3, *p < 0.05).

Later, we examined the phenotype of macrophages treated with DHA and Fer-1 in light of revealing the involved crucial mechanism of DHA-induced reprogramming in TAM. Similarly, the key proteins of the M1 phenotype such as CD86, iNOS, and GBP5 were investigated in the presence of DHA and Fer-1. As presented in Figures 8A–F, the biomarkers of the M1 phenotype were down-regulated when co-treated with DHA and Fer-1. Moreover, the inhibition of ferroptosis also attenuated the phagocytosis and antigen presentation functions of DHA-incubated TAM, as evidenced by the decreased endocytic latex beats and MHC-II expression in macrophage (Figures 8G,H). Altogether, evidence has emerged, suggesting a key role of ferroptosis in regulating the phenotypic remodeling in DHA-treated TAM.

### Reprogrammed M1-phenotypic macrophages exhibited prominent anti-cancer efficacy

**FIGURE 9:**
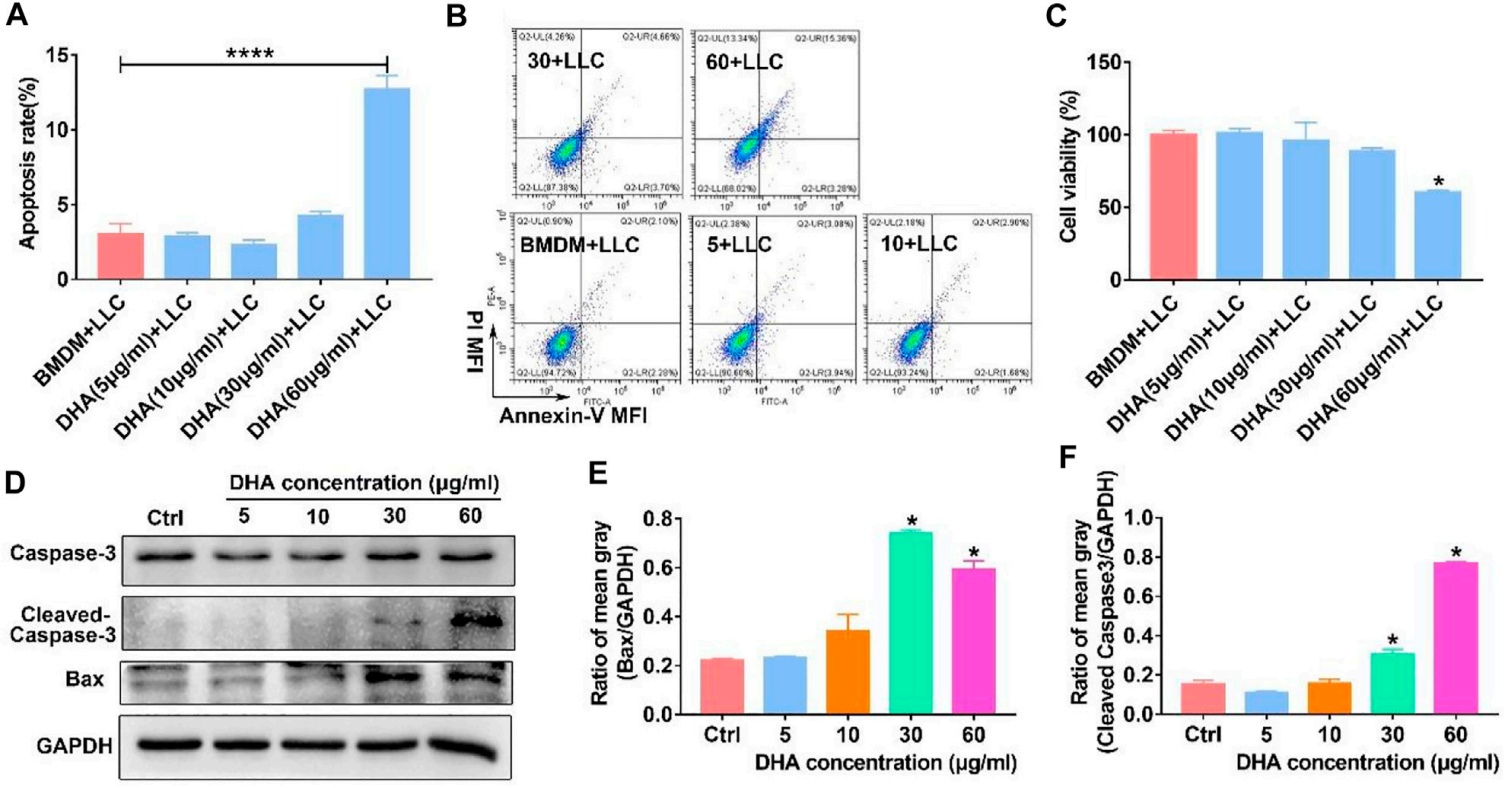
M1 phenotypic macrophages induced by DHA-facilitated apoptosis and of lung cancer cells. TAM were treated with DHA for 18 h, then co-cultured with LLC for another 18–24 h. **(A,B)** The apoptosis rate of LLC was measured through Annexin-V/PI double staining and flow cytometry. **(C)** The viability of LLC was assayed by CCK-8. **(D–F)** The expression of Bax and Caspase-3 in LLC, which indicated the apoptosis levels, was detected using WB. The mean gray of bands was quantitatively analyzed. Values were means ± SD (*n* = 3, *p < 0.05, ****p < 0.0001 which was compared with the control group).

The reprogrammed M1-phenotypic macrophages should have the ability of anti-tumor. To test the anti-tumor efficacy of DHA-treated TAM, Lewis lung cancer cells (LLC) were co-cultured with TAM that received DHA treatment. As expected, the apoptosis rate of LLC increased when co-cultured with TAM treated by DHA (Figures 9A,B). Furthermore, the viability of LLC decreased in the presence of DHA-treated TAM (Figure 9C). At the same time, the expression of Bax and Caspase-3 in LLC was reinforced with the addition of DHA-treated TAM (Figures 9D–F). These data were crucial proof that DHA-mediated reprogramming of TAM boosted anti-tumor immunotherapy.

## Discussion and conclusion

**FIGURE 10:**
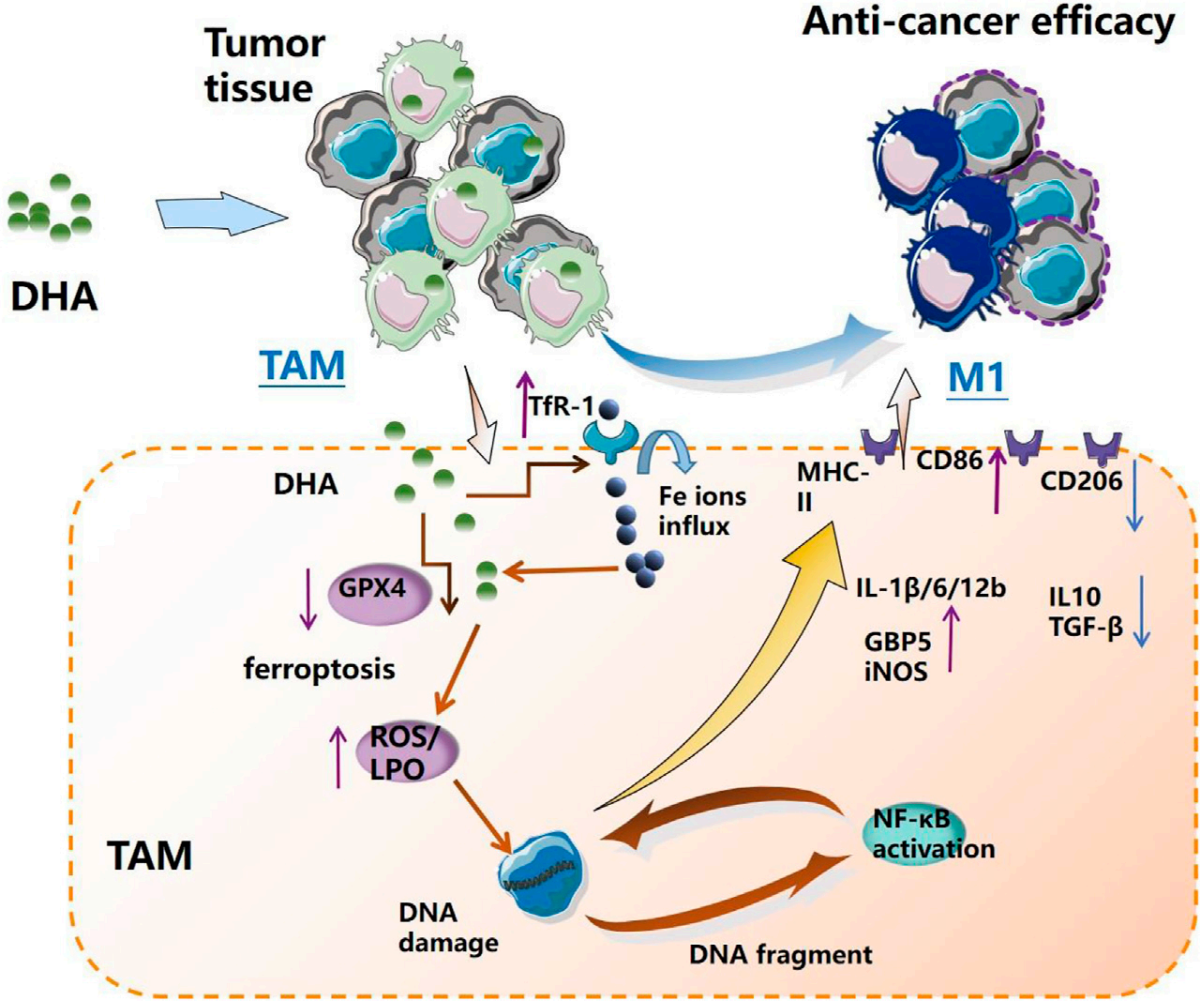
Schematic diagram of the mechanism of DHA-mediated reprogramming of TAM through ferroptosis. DHA-triggered ferroptosis of TAM generates ROS/LPO, leading to DNA damage, which activates downstream NF-κB to remodel TAM to the M1 phenotype. The reprogrammed macrophages possess an anti-tumor effect.

In the present work, DHA acted as a ferroptosis inducer to enrich ROS and LPO in TAM. LPO could damage DNA with broken fragments that activate the NF-κB molecule, thereby driving the inflammatory response in TAM, which reprogrammed macrophages into the pro-inflammatory M1 phenotype. The remodeled M1 macrophages could promote apoptosis of lung cancer cells *in vitro* (Figure 10).

The effect of DHA on inducing ferroptosis has been shown in the work of some scholars and in our previous study (Han et al., 2022). Triggering of ferroptosis requires both enrichment of iron ions and inhibition of the intracellular reducing system (Ye et al., 2020). However, the basal content of iron ions actually varies greatly among different cell types. In our work, the content of iron ions was also shown to be much higher in lung cancer cells than in TAM (Figure 3A). That is why the same concentration of DHA treatment of lung cancer cells caused a significant decrease in their viability (Han et al., 2022) while having a minor effect on TAM viability. In addition, this further leads us to understand the reason why DHA selectively kills tumors with low toxic side effects. Therefore, DHA played a dual role in the fight against lung cancer by directly destroying tumor cells and regulating the immune microenvironment. The fact that the present work has not explored the *in vivo* efficacy of DHA was a limitation. In general, the efficacy of *in vivo* studies is the overall result of DHA action on the tumor microenvironment, which cannot be attributed to TAM phenotypic remodeling even if there is prominent efficacy. Therefore, *in vitro* validation of immune efficacy was conducted. As presented in Figure 9, TAM was first treated with DHA and then co-cultured with lung cancer cells (LLC). The results revealed that DHA-treated TAM could promote apoptosis of LLC. The design of this *in vitro* study can adequately circumvent the attribution deficiency in the *in vivo* study.

On the other hand, interestingly, iron ion concentrations differed dramatically in the macrophages but with different phenotypes. Accordingly, the concentration of iron ions is lower in macrophages of the M2 phenotype (Agoro et al., 2018), which is consistent with our work. In contrast, the majority of macrophages with a pro-inflammatory phenotype (M1 phenotype) had higher iron ion concentrations (Zhou et al., 2018). Nevertheless, prior studies have not elucidated the underlying mechanism of this phenomenon. Instead, our study confirmed that DHA initiated ferroptosis by up-regulating TfR1 in combination with the inhibition of GPX4. In turn, the product of ferroptosis, LPO, further activated NF-κB by damaging DNA, thereby contributing to the polarization of macrophages toward the M1 phenotype. In the present study, an intrinsic link between elevated iron ions and macrophage phenotypic remodeling was established thereupon.

How DNA activation of NF-κB was caused by DHA-triggered ferroptosis of TAM was not highlighted in the present study. Nevertheless, the DNA double-strand break (DDSB) and DNA damage response (DDR) were observed in DHA-treated TAM (Figure 5). Recent studies have demonstrated that broken fragments of DNA released into the cytoplasm can activate the sensor molecule-STING, which is an important signal for anti-tumor immunity (Hopfner and Hornung, 2020; Ma et al., 2020; Reisländer et al., 2020). STING activation can activate its downstream NF-κB molecules, which we mentioned in our study of a photodynamic effect that remodels macrophages to the M1 phenotype (Sun et al., 2020; Yum et al., 2021). But the photodynamic effect is through ROS-mediated DNA damage, while DHA damages DNA by way of LPO generated by ferroptosis. Both are actually inextricably intertwined, but with subtle differences, which could serve as an approaching point for future research.

In conclusion, this work demonstrates that DHA-triggered ferroptosis of TAM results in DNA damage, which could activate downstream NF-κB to remodel TAM to the M1 phenotype (Figure 10), providing a novel strategy for anti-lung cancer immunotherapy. This study offers a novel theoretical basis for the use of traditional Chinese medicine monomers (such as DHA) to regulate the anti-tumor immune response, as well as a new therapeutic approach for TAM phenotype remodeling.

## Data Availability

The original contributions presented in the study are included in the article/Supplementary Material; further inquiries can be directed to the corresponding authors.
