# Supplementary material for: Dihydroartemisinin remodels macrophage into an M1 phenotype via ferroptosis-mediated DNA damage
Source: Front Pharmacol. 2022 Aug 11;13:949835. doi: 10.3389/fphar.2022.949835 (PMC9403990; doi:10.3389/fphar.2022.949835)
Supplement: Supplementary file 1 [file DataSheet1.docx]

**Supplementary materials**

**
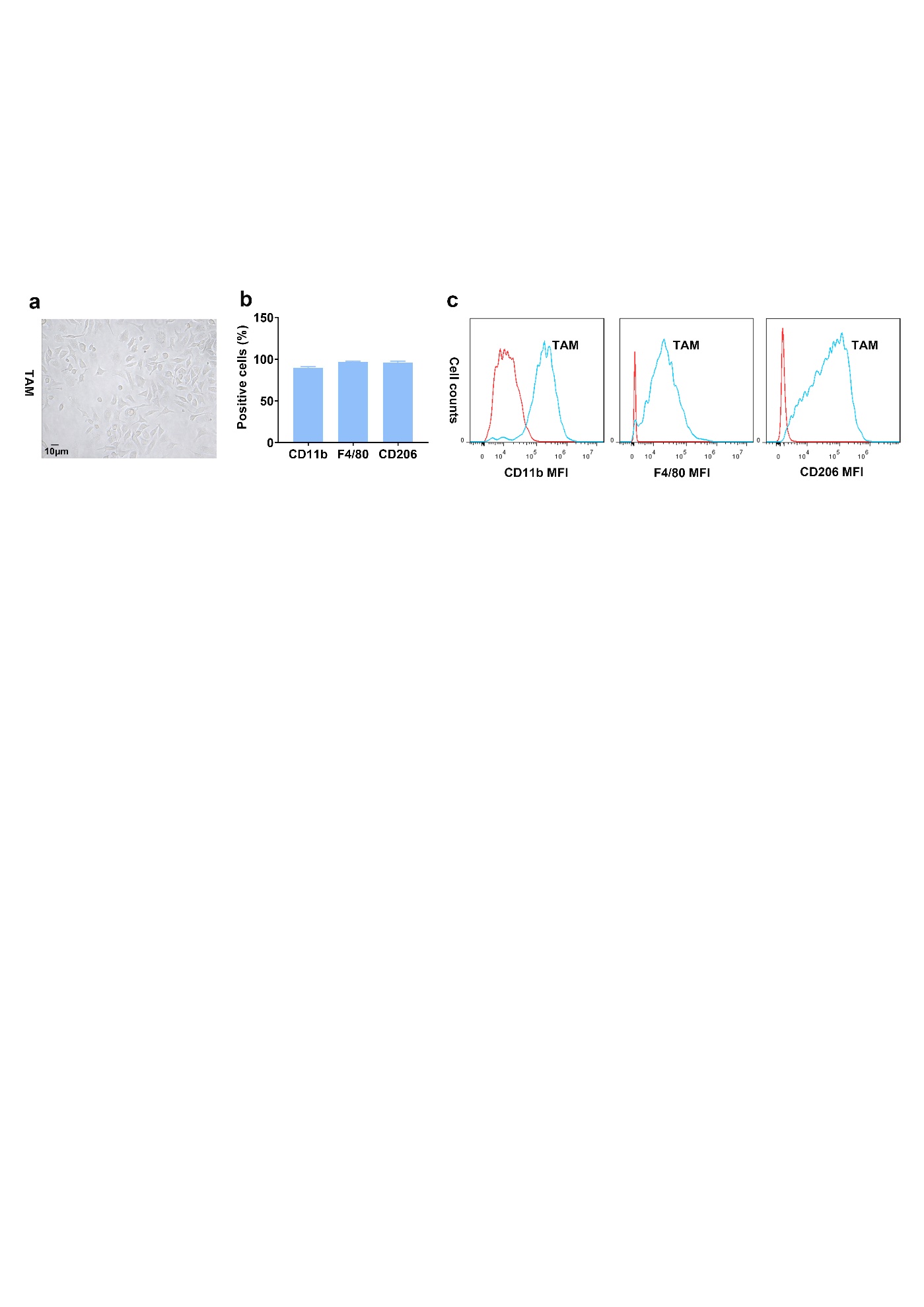
**

**Fig. S1. The identification of TAM from mouse bone marrow derived cells. A:** The morphology of TAM detected by microscopy. **B-C:** The TAM were identified through measurement of biomarkers (CD11b, F4/80 and CD206). Values were means ± SD (n=3, * p < 0.05).

**
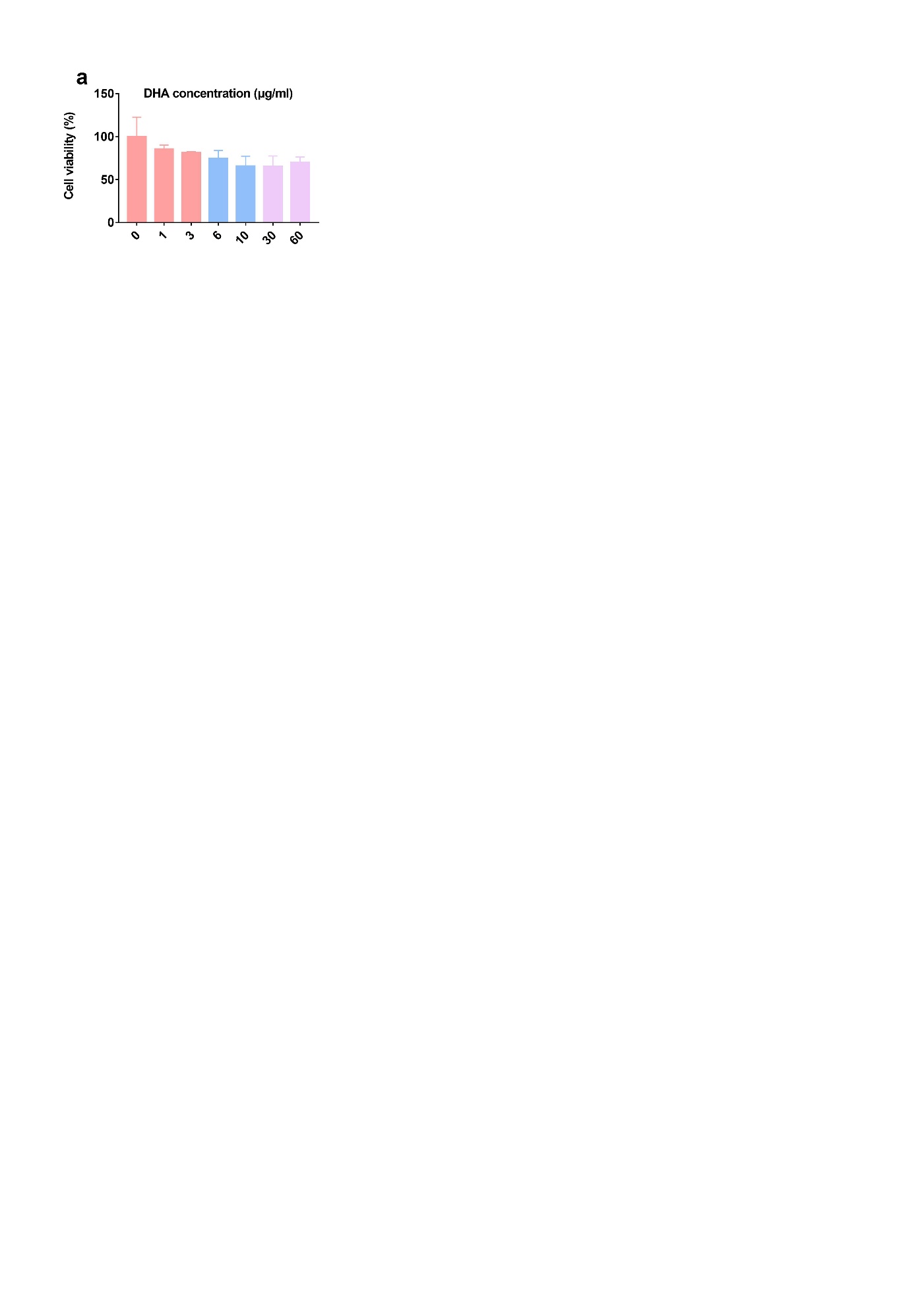
**

**Fig. S2. The viability of TAM varied little in response to DHA treatment.** TAM were treated with DHA. The cell viability was detected with CCK-8. Values were means ± SD (n=3, * p < 0.05).

**
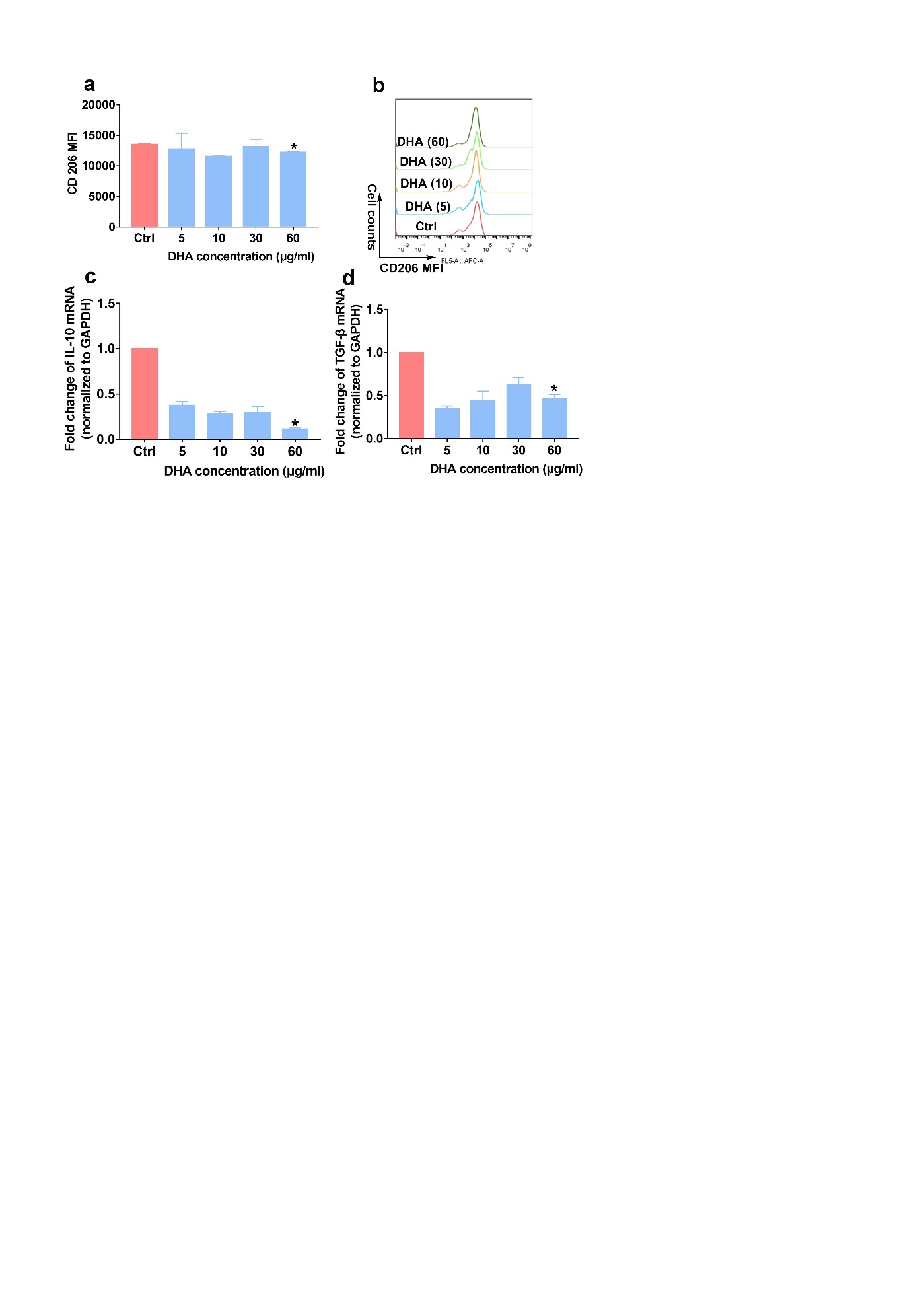
**

**Fig. S3. DHA re-polarized TAM from M2 phenotype. a-b:** CD206 membrane expression of TAM was measured by flow cytometry. **c-d:** mRNA expression of IL-10 and TGF-β was detected using RT-PCR. Geometric means were used to quantify the MFI. Values were means ± SD (n=3, * p < 0.05, compared with ctrl group).

**
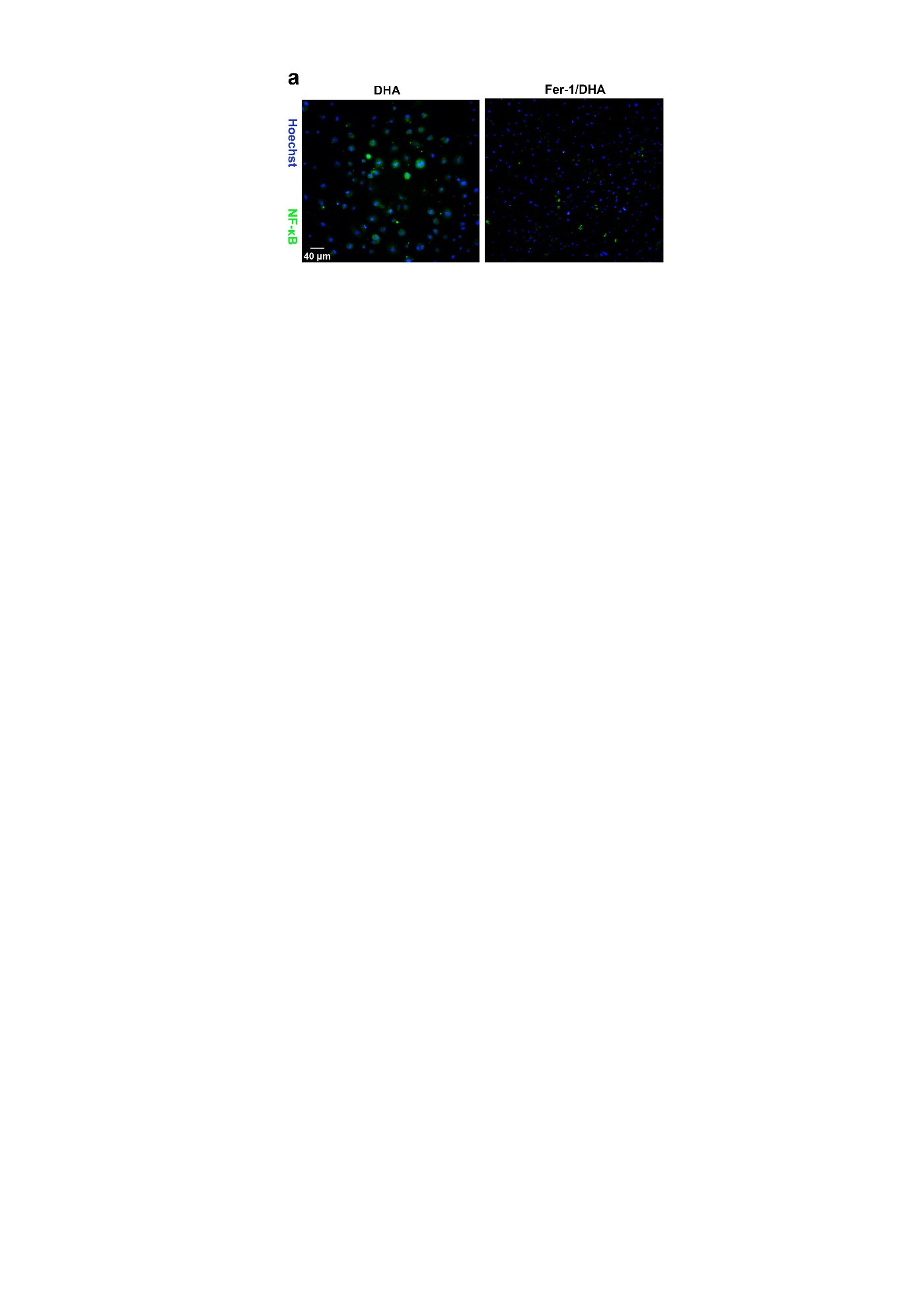
**

**Fig. S4. Co-addition of Fer-1 and DHA weakened nuclear translocation of NF-κB.** The green fluorescence came from NF-κB. Blue fluorescence came from nucleus.
